# Supervisory dyads’ communication and alignment regarding the use of workplace-based observations: a qualitative study in general practice residency

**DOI:** 10.1186/s12909-022-03395-7

**Published:** 2022-04-28

**Authors:** Laury P. J. W. M. de Jonge, Floor N. E. Minkels, Marjan J. B. Govaerts, Jean W. M. Muris, Anneke W. M. Kramer, Cees P. M. van der Vleuten, Angelique A. Timmerman

**Affiliations:** 1 Department of General Practice, Maastricht University, P.O. Box 616, 6200 MD Maastricht, The Netherlands; 2 Department of Educational Research and Development, Maastricht University, Maastricht, The Netherlands; 3 Department of Family Medicine, Leiden University, Leiden, The Netherlands

**Keywords:** Assessment, Workplace learning, Dialogue, Educational alliance, Feedback, Performance observation

## Abstract

**Background:**

In medical residency, performance observations are considered an important strategy to monitor competence development, provide feedback and warrant patient safety. The aim of this study was to gain insight into whether and how supervisor-resident dyads build a working repertoire regarding the use of observations, and how they discuss and align goals and approaches to observation in particular.

**Methods:**

We used a qualitative, social constructivist approach to explore if and how supervisory dyads work towards alignment of goals and preferred approaches to performance observations. We conducted semi-structured interviews with supervisor-resident dyads, performing a template analysis of the data thus obtained.

**Results:**

The supervisory dyads did not frequently communicate about the use of observations, except at the start of training and unless they were triggered by internal or external factors. Their working repertoire regarding the use of observations seemed to be primarily driven by patient safety goals and institutional assessment requirements rather than by providing developmental feedback. Although intended as formative, the institutional test was perceived as summative by supervisors and residents, and led to teaching to the test rather than educating for purposes of competence development.

**Conclusions:**

To unlock the full educational potential of performance observations, and to foster the development of an educational alliance, it is essential that supervisory dyads and the training institute communicate clearly about these observations and the role of assessment practices of- and for learning, in order to align their goals and respective approaches.

**Supplementary Information:**

The online version contains supplementary material available at 10.1186/s12909-022-03395-7.

## Background

In medical residency training, observation of residents’ clinical performance is an important strategy to support the provision of feedback for learning, enable high-stakes decision-making about competence development and to guarantee high-quality patient care [1–5]. Continuous use of observations throughout medical training programmes is therefore strongly recommended [6, 7].

Despite its importance, however, observing resident performance does not seem to be habitual practice [7–10]. The literature has suggested that several factors may undermine the use of observations in residency training, such as tensions between patient safety and the need for residents to learn from their mistakes, as well as between direct supervision needs and residents’ quest for autonomy [1, 10–16].

Hence, supervisors and residents may have similar but also diverging goals and preferential strategies for achieving these goals when engaging in observations of clinical performance. When misaligned, these differences may inadvertently impact on training outcomes. Indeed, it is increasingly recognised that effective performance observations require a supervisor-resident relationship that is primarily based on collaboration and trust [17, 18]. To foster learning in the workplace, moreover, such a relationship must be a two-way process in which clear communication, openness and agreement about goals and tasks are paramount [19, 20]. Drawing an analogy with a therapeutic working alliance, Telio et al. (2015) conceptualised the supervisor-resident relationship as an educational alliance in which the unity of goals, agreement on the approach to reach these goals and the perceived effectiveness of the supervisor-resident bond are fundamental [21]. If such an alliance is present, supervisors and residents will communicate their individual goals regarding performance observations and align their preferred approaches to reach these goals accordingly, potentially leading to shared understanding, the formulation of common goals, and co-construction of action plans. It is unknown, however, whether supervisory dyads in residency training have already taken up such practice and if so, how.

The aim of this study was therefore to gain insight into if and how supervisor-resident dyads work towards alignment of goals and approaches regarding the observation of residents’ clinical performance. These insights can contribute to our current knowledge about the formation of educational alliances in medical residency training and may serve to further optimise the use and effectiveness of observations in clinical workplace-based learning.

## Methods

### Setting

This study was conducted in the three-year general practice residency training programme at Maastricht University, the Netherlands. In the first and third years of this programme, residents spend 4 days per week in general practice and return to the training institute weekly for a 1-day release programme. In general practice, residents receive long-term and one-to-one supervision in the provision of patient care. The assessment programme used to evaluate residents’ competence development is based on the CanMEDS competency framework [22] and spans a variety of formative and summative assessment methods at all levels of Miller’s pyramid [23]. Single encounter assessments in the clinical workplace (based on direct or videotaped observations of clinical encounters or case-based discussions, for example) serve to document performance feedback in the resident’s portfolio. In addition to these workplace-based assessments, the portfolio also includes information from standardised institutional assessments, including obligatory knowledge tests (twice a year) and a consultation test (mid-year in year 1 and 3, respectively). For this latter test, a designated assessor from the training institute assesses six self-selected, videotaped, real-life clinical encounters following a strict legal protocol. Although the primary purpose of the institutional assessments is to provide formative feedback, residents must follow a remedial programme if their performance is perceived to be below expectations.

### Participants and sampling

We selected participants from a pool of 24 supervisors who, at the time of data collection, were active as supervisors in our region. We used purposive sampling in order to include supervisor-resident dyads with differing years of supervisor experience and from differing years of training (Table 1). We excluded residents who had started their training programme only recently (< 3 months), because they had too little experience with the social process under scrutiny. Supervisors were contacted by email to invite them and their residents to participate in this study. Eight supervisor-resident dyads agreed to participate; residents were equally distributed in terms of gender (four male; four female) and year of training, their progress ranging from the 4th to the 12th month of the respective year of training. Supervisors’ experience ranged from 2 to10 years; six supervisors were male.

**Table 1:** Demographic information of participants in the semi-structured interviews

	Resident (male/female)	Year (month) in general practice residency training	Supervisor (male/female)	Years’ experience as a general practice supervisor
Dyad 1	Female	1 (10)	Male	5
Dyad 2	Male	1 (11)	Male	2
Dyad 3	Female	1 (5)	Male	4
Dyad 4	Male	3 (10)	Male	4
Dyad 5	Female	3 (12)	Female	7
Dyad 6	Female	3 (10)	Male	6
Dyad 7	Male	1 (5)	Male	10
Dyad 8	Male	3 (4)	Female	6

### Methodology and reflexivity

Our research team consisted of individuals with different areas of expertise and perspectives on processes related to assessment and learning in the clinical workplace. LJ is a general practitioner and educator; FM is a final year medical student with personal experience of being observed during clerkships; AT is a psychologist working at a general practice training programme; MG is a medical educator involved in assessment design; AK and JM are general practitioners with clinical supervisory experience; and CV is an educational psychologist. In addition, all researchers have a record of accomplishment in medical education research. We used a qualitative, social constructivist approach to explore if and how supervisory dyads work towards alignment of goals and approaches to observation of clinical performance. We recognised that the phenomenon under scrutiny is subject to multiple interpretations, depending on the position of the researcher, the participants and the research context [24, 25]. Yet, researchers’ knowledge of the study setting enabled them to provide depth of reflection and interpretation. Throughout iterative cycles of data collection and analysis, they acknowledged their individual perspectives and stances. They did so by regularly discussing their findings from individual data collection and analysis within the research team and by exploring how their personal interpretations contributed to making meaning of the data.

### Data collection and procedure

We collected our data from a series of semi-structured interviews with the resident, the supervisor and the supervisor-resident dyad for each practice, respectively. In the first two individual interviews, we explored supervisors’ and residents’ preferences and the way they perceived individual goals and approaches regarding the use of observations. In the final interview, we asked the dyad to summarise their individual and perceived common goals and approaches to the use of observations, before inviting them to reflect on their viewpoints on if and how they worked towards alignment during residency training. At the end of the dyad interview, one of the interviewers briefly summarised and reflected on findings and invited the participants to comment on these reflections.

We based our interview guide (Additional file 1) on relevant literature about supervisor and resident perceptions regarding the use of observations in medical residency training [1, 4, 10, 11, 13, 15, 16, 26] as well as on research on workplace interactions between residents and supervisors [19, 21, 27–29]. All team members were individually invited to give feedback and uncover their initial views and assumptions about possible themes in the interview guide. After that, the first author incorporated feedback into a revised version of the interview guide which was subsequently discussed in the research team. To prevent the use of restrictive or guiding questions and optimise the informational value of the data collected, we tested the interview guide in one practice setting. As this test only led to minor revisions to our interview guide, we included the data from these interviews in the final data set. Audio recordings of all interviews were transcribed verbatim and anonymised prior to analysis. Two researchers (FM, LJ) conducted all interviews together from December 2018–March 2019; the set of three interviews per practice lasted between 80 and 120 minutes in total.

### Data analysis

We performed a template analysis of our data, for which we iteratively created a series of templates consisting of hierarchically structured schemas of coded themes [30–32]. Our initial coding framework (initial template) was based on the themes of our interview guide. This template was then complemented with themes resulting from a preliminary analysis of the first two series of interviews. The resulting template served to summarise whether and how supervisor and resident communicated and aligned their individual goals and preferred approaches regarding observations. LJ and FM then modified the template based on continued coding and frequent discussions with the research team. Throughout data-analysis, we combined a deductive and inductive approach by complementing and refining our a priori coding framework with salient themes that were identified in our interviews. To identify additional themes and to prevent early narrowing of ideas, an additional researcher (AT) coded the transcripts pertaining to the interviews with the fourth dyad. Throughout the coding process, LJ, FM, AT and MG identified and discussed relationships between the themes within and across dyads to develop a final template. Every 6 weeks, we discussed our findings within the complete research team to assess the authenticity, comprehensibility and completeness of the evolving template. To identify additional themes and to prevent early narrowing of ideas, an additional researcher (AT) coded the transcripts pertaining to the interviews with the fourth dyad. Throughout the coding process, LJ, FM, AT and MG identified and discussed relationships between the themes within and across dyads to develop a final template. After six interview series had been coded (18 interviews), we concluded that the template sufficiently covered the themes present in the interview data as we could not identify any new themes. To confirm the final template (Additional file 2), we coded the two series of interviews remaining. LJ and FM applied this final template to the full data set and discussed the findings with the research team until they reached consensus about the definitive interpretation. We used NVivo software, version 12 (QSR International Pty Ltd.), to facilitate the qualitative coding of interviews [33].

## Results

We should point out that all supervisors in this study had the practice of observing the clinical performance of their residents. In the next sections, we will first set out in detail how dyads reached a working repertoire on the use of observations over time. We will then zoom in on the educational alliance, and the role of communication and alignment in the dyad’s creation of a working repertoire. To support our interpretation of the data, we will also include representative quotes from supervisors (S) and residents (R).

### Supervisor preferences prevail at the start of training

At the start of training, all supervisors had an introductory learning conversation with their residents to provide general information on their supervision practice. One of the items up for discussion was how and on which occasions supervisors preferred to use performance observations. This communication was explicit, leaving no question as to its meaning or intent, and was recognised as such by both supervisor and resident. As a consequence, it often received the formal status of an agreement and concomitant plan of action:

When we agreed in the learning conversation that we had to perform one observation per week, we just asked the medical receptionist to plan a weekly observation in both our agendas. Otherwise, it remains tempting to postpone to the next day or even the next week. (S5)

Although supervisors expected the resident to observe some of their consultations in the first week, they gradually swapped roles, so that supervisors were the ones observing residents’ clinical performance. Both supervisor and resident shared the view, albeit implicitly, that this approach primarily served to warrant safe patient care:

‘When I start with a new resident, the primary goal for me to observe is to acquire a sense of safety, peace of mind that you can leave patient care to that resident’. (S6)

‘I think that, specifically at the start of the year, he [S6] wants to know: “what kind of person am I exposing my patients to?’. (R6)

At the start of the year, supervisors typically expressed a preference for videotaped patient consultations, often leaving the initiative to plan direct observations of clinical performance to the resident. Many residents, however, agreed with their supervisors that video observations were ‘a good preparation for the consultation test’. In fact, supervisors and residents mainly used these observations to prepare residents for this institutional test as well as to warrant patient safety, paying scant attention to the opportunity they offered to provide or receive feedback for personalised learning.

### Scarce explicit communication during training

After a few weeks, explicit communication about the goals and approaches to observations mostly disappeared from the agenda of the learning conversation. In the interviews with dyads, supervisors and residents mentioned that they did not specifically discuss the goals or preferred approaches to observations: they were more of *‘a habit that crept in’* (dyad 5) or ‘*just went that way’* (dyad 8) and it *‘felt natural not to be explicit upon’* (dyad 1). When they did communicate explicitly about observations, the focus was often on practical aspects of the working repertoire that needed attention, such as the planning of observations, whether to use a standardised rating scale to better prepare for the consultation test, or, as the next quote illustrates, a request to be observed when performing specific procedures:

‘ … procedures, like injections, nail extractions, insertion of intrauterine devices’. (R6)

‘Yes, indeed, intra-articular injections’. (S6)

‘And an update now and then of my checklist, so that I know: I did that, and you [the supervisor] observed me while I was performing these procedures’. (R6)

However infrequent, explicit communication about observations during training was often triggered by internal and external factors (i.e. originating from inside or outside the supervisory dyad). Stagnation of expected competence development, as perceived by the supervisor and/or the resident, for instance, could act as an important internal trigger. In such cases and acting in the best interests of both patients and residents, supervisors would initiate observations ‘to assess what is going on’ (S5). By doing so, they made sure that substandard performance was met with adequate measures, as the next quote illustrates:

It depends on how things are going. If I detect shortcomings, or recurring points of attention, then I will say, ‘Hey, from now on I want us to perform an observation every week’. (S1)

Residents, in their turn, could actively seek more guidance in the form of supervisory observations when they felt they were lagging behind:

When I’m in doubt about my approach to a procedure, or if I’m persistently doing something wrong, it would not be okay if that was not said or seen and I would keep on doing this. Then I would also ask him [S2] to observe me more often. (R2)

In addition to these internal triggers, we found that the obligatory institutional consultation test acted as a strong external trigger to initiate a discussion about the dyad’s goals and approaches to observations. To prepare for this test so that the resident would perform well, dyads primarily resorted to video observations and were mostly concerned with ‘ticking off’ items on the standardised rating scale during their learning conversations:

In the end, the test is all about having your video recordings done. Moreover, I am just honest about that, they would like to see the MAAS-Global [standardised rating scale] skills, so you are going to act differently to get that ticked off. … After the test, we discussed that you don’t have to show everything in a single patient consultation, and she [S8] helped me not to get fixated on that. (R8)

As soon as the resident had passed the consultation test, however, dyads’ reasons for engaging in observations often shifted from being performance-oriented to being more learning-oriented. Consequently, dyads adopted a more tailored approach to observations with, for example, the aim to support the resident’s development of communication skills. In almost all dyads, passing the test led to a reduced uptake of video observations in favour of direct observations, as the following quote illustrates:

Well, with the consultation test in mind, we watched many videotaped consultations, of course also because that serves as a moment of assessment. After that, it just became less. Then, for instance, we discussed that we preferred more mutual observations during house calls. (R2)

### The role of communication in achieving alignment within the dyad

As reported previously, explicit communication within the dyad about the goals of observations and respective approaches was mostly limited to the start of the training year or triggered by specific internal and/or external factors. Yet, all dyads felt mutually responsible for performing observations:

‘I think it is mainly the responsibility of the resident to discuss the use of observations, as it is his training. However, I will take over if he doesn’t, because I also consider them an essential monitoring tool’. (S4)

‘We are in residency together; as a resident you have your responsibility because it’s your learning process. However, the supervisor must of course also invest time and energy to facilitate observations, so we are both responsible’. (R5)

Over time, dyads typically achieved a working repertoire without explicit communication, leaving individual preferences undiscussed. In the next dialogue from a dyad interview, the resident and supervisor had conflicting goals, which, however, they did not explicitly communicate: while the resident avoided observations because he wanted to learn to work autonomously, the supervisor-initiated observations with the aim to monitor the resident’s competence development. In the end, however, the resident did appreciate this initiative for its learning value:

R4: One of my learning goals was to work more autonomously during evening shifts; therefore, I stopped asking my supervisor to join me. Yet, recently I noticed that he [the supervisor] planned working in shifts together again. …

S4: Well, indeed, because these were your last shifts, and I wanted to dot the i's and cross the t's.

R4: I learnt a lot from doing shifts independently, but together, you can gain new insights, particularly from being observed. Therefore, it is this mixture, that arises gradually, and not from a clear plan; it happens automatically.

Although the dyad above did not explicitly communicate their reasons for wanting (or not wanting) to perform observations, they did manage to create an effective working repertoire regarding the use of observations. The next interview with another dyad, by contrast, illustrates that leaving individual goals unsaid could also lead to a mismatch in terms of the desired frequency of observations. In this case, the resident felt uncomfortable about the level of autonomy assigned to her and therefore would have liked to be observed more frequently to protect her patients:

R1: I acted very autonomously, but I thought ‘is he aware of what I’m doing, and is that safe for the patients?’ I would feel more comfortable if he [the supervisor] would see what I did, that I knew that he could really trust me with what I did.

S1: I didn’t know that.

When dyads did specifically discuss their preferred method of observation, however, this did not always lead to alignment. Power dynamics in the supervisor-resident relationship, for example, potentially influenced decisions about the working repertoire and actual agreements on goals and approaches to performance observations, as the following dyad interview illustrates:

R7: Personally, I would like to be observed directly more frequently, to taste the atmosphere, while you [S7] strongly prefer videos.

S7: It’s simply too time-consuming, and I think I can actually see enough on video. We talked about it, and I think we found middle ground, but it’s more my middle ground than your middle ground, so to say.

In the individual interview with this particular resident, the misalignment referred to above caused the resident to feel he missed out on specific learning opportunities that direct observations had to offer:

A video is good for a consultation test and it is sometimes good to discuss with your supervisor, but I think direct observation is just more instructive, as it focuses on the whole picture and not on small details. (R7)

Finally, all dyads mentioned spontaneously that it could be beneficial if they adopted a more proactive approach to discussing the use of performance observations for learning purposes, even when patient safety was warranted and things were going well:

‘Even when things are running smoothly, you still might ask questions or do things differently, so that you could get more out of observations, for learning purposes’. (R6)

Looking back, you adopt a more reactive approach when you think things are running well. And that can be a pitfall. So when you would, for example, discuss more regularly about how the resident prefers to receive feedback, you might smooth off the rough edges to avoid a mismatch in feedback style. (S5)

## Discussion

The present study aimed to investigate if and how supervisor-resident dyads work towards alignment of their goals and preferred approaches regarding observations of clinical performance in general practice training. It seemed that dyads mainly engaged in such observations to guard patient safety and to prepare for institutional assessment rather than to provide the resident with developmental feedback. It was uncommon for them to communicate explicitly about the goals and preferred approaches to these observations, except at the start of training and unless they were triggered by internal or external factors. Nevertheless, supervisory dyads did manage to build a working repertoire regarding their use during the training year. The fact that dyads they did not consistently, explicitly and constructively, communicate about the goals of observations, however, might impair the establishment of a nurturing formative growth relationship in which observations are used for continuous learning and competence development.

Using observations as a supervisory strategy to provide safe patient care resonates with recent literature. Brown et al., for instance, found that ensuring safe patient care was the primary goal of help-seeking supervisory encounters in general practice training [34]. Of note, moreover, is that the dyads in their study used three different supervisory strategies to achieve their goals: 1) they prioritised patient care and supervisor modelling, 2) they focused on residents’ learning opportunities, and 3) they developed resident independence by leaving the initiative to ask for supervision with the resident [34]. These strategies bear a similarity to the approaches the dyads in our study adopted. Confirming research in similar settings, our study suggests that bilateral observations at the start of residency gave residents the opportunity to observe their supervisors’ performance, while at the same time informing supervisors of the level of supervision needed to warrant patient safety [26, 35]. When safety was warranted, the initiative to ask for observations was left to the residents, thereby supporting their development towards autonomy.

Unlike Brown et al.’s second supervisory strategy, however, the dyads in our study hardly engaged in observations with the aim to provide the resident with developmental feedback. Recent research by Watling et al. (2016) has indeed suggested that the strive for efficiency can dissuade both supervisor and resident from conducting observations for educational purposes [13]. What our study adds is that, apart from patient safety being dyads’ chief preoccupation, specific external demands, such as the institutional consultation test, could also divert attention away from residents’ individual learning as the focus of observations. When we consider the literature on goal orientation, we find that individuals can be driven by either a need to *prove* their competence (performance goals) or by a desire to identify learning opportunities to *improve* their competence (mastery goals) [36]. It is plausible to assume that, initially, the dyads in our study were essentially performance-oriented, as their observations mainly served to tick off items pertinent to the standardised institutional consultation test, even though the primary aim of this test was to provide developmental feedback. After passing the test, however, the dyads gradually started to look for learning opportunities for the resident, implying a shift to a more mastery-oriented (i.e. learning-oriented) use of observations.

The above findings confirm recent research suggesting that external assessments that do not take place in the clinical workplace (such as the consultation test) might be perceived as summative, thereby inducing performance-oriented teaching and learning for the test [37]. Only when residents and supervisors perceive assessments -such as performance observations- as formative, they may shift their focus from performance to learning [1, 14, 38–41].

In a similar vein, the lack of communication about the goals and approaches of performance observations identified in our study seems to be common practice in clinical supervision. In their study on variability in supervisory practice, Goldszmidt et al. (2015), for instance, found that supervisory approaches were all of a tacit nature: although supervisors used various approaches to warrant patient safety, provide feedback and simultaneously prepare their residents for institutional assessment, variations in supervisory approach were consistently not discussed within the supervisory dyad [42]. Finally, in their systematic review of supervisory relationships in general practice training, Jackson et al. (2019), too, concluded that dyads failed to explicitly share their expectations regarding supervisory goals, tasks and roles, flagging this as a key area for improving communication within the supervisory dyad, and, with that, the quality of learning and supervision [43].

### Strengths and limitations

In this study, we used template analysis to build on and refine existing theoretical concepts regarding the use of performance observations in residency training. A strength of this study is that we adopted a social constructivist approach that was well suited to our exploratory research question. Our semi-structured, in-depth interviews enabled us to identify the individual perspectives and preferences of supervisors and residents regarding the use of performance observations, and to gain insight into if and how they worked towards alignment when using this supervisory strategy.

Some limitations to our study, however, also need addressing. Our findings were entirely based on self-report data from participants rather than on our own observations of participants’ communication and behaviour in clinical practice. To avoid conflicts, participants may have responded in a socially desirable way, especially in the dyad interviews. Not only did the dyad interviews facilitate our inventory of participants’ preferences regarding goals and approaches, they also inadvertently sparked a discussion between residents and supervisors about individual perspectives and steps to achieve alignment, which potentially affected our results. During our interviews, the impact of power in hierarchical relationships emerged as an important finding. However, we did not purposively explore the role of additional factors that might affect communication within supervisory dyads nor came related themes (for example, gender-typical communication patterns) to surface during the analysis of our data.

Finally, our small sample size as well as the long-term and one-to-one supervisor-resident relationship that is typical of our setting may limit the generalisability of our results, for instance to hospital settings where the composition of supervisor-resident dyads changes continuously.

### Implications for educational practice and research

In addition to confirming previous study findings, our study seems to suggest that there might be a mismatch between the way supervisory dyads use observations and the purposes espoused and communicated by educators and/or national bodies. Hence, our findings are consistent with socio-constructivist views on assessment that explicitly acknowledge the role of assessment users’ views, beliefs, values and attitudes in shaping assessment behaviours [44, 45]. Supervisor-resident dyads must be aware of the ways they communicate about the use of performance observations in residency training, of factors that are known to affect communication and decision-making -including power differences and gender communication differences-, and their potential impact on the creation of a shared working repertoire [46, 47]. To unlock the full potential of observations, working towards an effective educational alliance requires awareness of and transparency about the individual goals and preferential approaches of both supervisor and resident, in equal partnerships [48, 49]. Residents must thus feel free to be open about their learning needs and that supervisors must explicitly discuss their agenda and role. This implies that we may need to pay more attention to empowering residents to speak up in inherently hierarchical supervisory relationships as well as to supporting supervisors in creation of trustful collaborative relationships with their residents. Future research may elucidate on these power differences and other parameters (for example differences in gender and ethnic backgrounds) that may affect communication interactions within supervisory dyads.

Our findings clearly demonstrate that the assessment programme in which observations are embedded may influence their perceived purpose and actual use. Therefore, to foster learning from workplace observations, educators must be aware of the impact of additional assessment requirements embedded in assessment programmes, and clearly and continuously articulate and align assessment goals.

In our study, approaches to observations were typically performance-driven, that is, mainly serving to avoid unsafe medical practice and to prepare for an institutional test. At the same time, using observations for learning and ongoing improvement received scant attention. Embedding facilitated discussion and alignment of the various goals and approaches to observations in learning conversations may be a promising avenue to improve their effectiveness in workplace learning. Training supervisors and residents to balance the development of residents’ professional autonomy while performing observations could be an important strategy to warrant both patient care and the provision of feedback for learning.

In all medical residency settings, observations of clinical performance are generally considered an important source of feedback for learning, even in in the absence of negative events [1, 16, 50]. Additional research on the formation of working repertoires for the use of observations in other clinical settings may therefore further our understanding of how to enhance educational alliances in residency training.

## Conclusion

The use of observations of clinical performance to guard patient safety and to prepare for institutional standardized tests seems self-evident in general practice residency, resulting in supervisory dyads adopting working repertoires that best serve these (implicitly shared) goals. However, testing, even when intended to be formative, may be perceived as summative by supervisors and residents, and lead to teaching to the test rather than engaging in observations for the purposes of personalised learning. To unlock the full educational potential of observations in workplace learning, awareness, ongoing communication and alignment of goals and approaches to observation, not only within the supervisory dyad but also between the dyad and the training institute are therefore key.

## Supplementary Information

**Additional file 1.** Guide for the interviews with the resident, supervisor and supervisor-resident dyad.

**Additional file 2.** Overview of final template for data analysis.

## Data Availability

The datasets generated and/or analysed during the current study are available in the DataverseNL repository, 10.34894/MKLCNI.

## Abbreviations

CanMEDS: Canadian Medical Education Directives for Specialists
