# Supplementary material for: Supervisory dyads’ communication and alignment regarding the use of workplace-based observations: a qualitative study in general practice residency
Source: BMC Med Educ. 2022 Apr 28;22:330. doi: 10.1186/s12909-022-03395-7 (PMC9052511; doi:10.1186/s12909-022-03395-7)
Supplement: Supplementary file 1 — Additional file 1. Guide for the interviews with the resident, supervisor and supervisor-resident dyad. [file 12909_2022_3395_MOESM1_ESM.docx]

**Additional file 1.** **Guide for the interviews with the resident, supervisor and supervisor-resident dyad**

**Individual interviews with the supervisor and resident (parts I and II):**

- Definition and various methods of observation

Perceptions and actual behaviour regarding:

- tasks, goals and strategies regarding observations
- taking the initiative and responsibility to perform observations
- dealing with barriers to and facilitators of observations

**Interview with supervisor-resident dyad (part III)**

Perceptions and actual behaviour regarding:

- communication about the use of observations and reaching agreement
- the approach to using observations over time
- different perspectives between supervisor and resident when establishing an approach regarding the use of observations
- agreement on establishing a working repertoire

**Part I. Interview with resident**

*We used the following probing questions, after which the respondents were requested to elaborate on their answers or provide examples to illustrate them:*

1. [Definition of observation, methods and use of observations during training]

•         *Could you define what observation means to you during workplace-based learning?*

•         *What methods of observation do you know?*

•         *What methods of observation are used to observe your performance during your training?*

•         *In what clinical settings are you observed during your training?*

2. [Initiative for observations, planning, and frequency of observations]

*Initiative for observations*

•        *Who takes the initiative to perform observations during your training?*

•         *Whose responsibility do you think it is to ensure that observations take place during your training?*

•         *How do you experience taking the initiative yourself to be observed during your training?*

•         *How do you experience it when your supervisor takes the initiative?*

*Planning and frequency of observations, including perception*

•         *When and how often are you observed during your training?*

•         *Are there any scheduled moments that you are observed?*

•         *Do observations also take place spontaneously during your training?*

•         *Is there a difference in the frequency of observations during the training year?*

•         *Is it common for you to be observed during your training?*

3. [Goals and merits of observations for the resident]

•       *What is or are your goal(s) when you are observed?*

•        *Do you formulate a learning question when you are observed?*

•        *What are merits of observations for you?*

•       *Do you receive feedback from observations?*

  4. [Goals and merits of observations for the supervisor as estimated by the resident]

•        *Is it clear to you what the goals of your supervisor are when he or she performs observations?*

•        *What do you think are merits of observations for your supervisor?*

5. [Barriers and facilitators regarding the performance of observations as perceived by the resident]

•       *Are there any barriers for you to be observed during your training?*

•        *Have you ever had a situation where you did not want to be observed? How do you feel about working and learning independently in clinical practice? Does this prevent or stimulate you to be observed?*

•       *How do you experience your supervisor observing you during your training?*

•       *How do you tend to see an observation, as an assessment or a learning opportunity?*

•       *Are there facilitators that promote or make it easier for you to perform observations during your training?*

•      *How does the learning climate play a role in making observations during your training?*

[Barriers and facilitators for the supervisor as estimated by the resident]

•       *Do you think the supervisor experiences any barriers to make observations during your training?*

•       *How do you perceive that your supervisor experiences being present when observing your clinical encounters with a patient?*

•         *Does the way in which you as a resident handle observations have an effect on your supervisor to observe you or not during your training?*

•        *Are there factors that promote or make it easier for your supervisor to perform observations during your training, from your perspective?*

6. [Effects of performing observations on supervisor-resident relationship]

•         *Does performing observations affect the relationship between you and your supervisor during your training?*

**Part II. Interview with supervisor**

*We used the following probing questions, after which the respondents were requested to elaborate on their answers or provide examples to illustrate them.*

1. [Definition of observation, methods and use of observations during training]

•         *Could you define what observations mean to you?*

•         *What methods of observation do you know?*

•         *What methods of observation do you use to observe the resident during training?*

•        *In what settings do you observe during training?*

2. [Initiative for observations, planning, and frequency of observations]

*Initiative for observations*

•      *Who takes the initiative to perform observations?*

•      *Whose responsibility do you think it is to ensure that observations take place during training?*

•       *How do you experience taking the initiative to observe?*

•       *How do you experience it when the resident takes the initiative?*

*Planning and frequency of observations, including perception*

•         *When and how often do you observe the resident during training?*

•         *Are there any scheduled moments that you perform observations?*

•         *Do you also observe the resident spontaneously?*

•         *Is there a difference in the frequency of observations during the training year?*

•         *Is it common for you to observe the resident during their training?*

3. [Goals and merits of observations for the supervisor

•       *What is or are your goal(s) when you perform an observation?*

•        *Does the resident formulate a learning question when he/she is going to be observed?*

•        *What are for you the merits of observations?*

•       *Do you provide feedback after observations?*

  4. [Goals and merits of observations for the resident as estimated by the supervisor]

•        *Is it clear to you what the goals or objectives of your resident are when you observe him/her?*

•        *What do you think are merits of observations for your resident?*

5. [Barriers and facilitators regarding the performance of observations as perceived by the supervisor]

•       *Do you experience any barriers to perform observations?*

•        *Have you ever had a situation where you did not want to, or were not able to observe your resident?*

•        *What is your perception of residents working and learning independently in practice? Does this prevent you from observing your resident?*

•       *How do you experience observing your resident during training?*

•       *Do you tend to see an observation as an assessment or as serving another purpose?*

•       *Are there factors that promote or make it easier for you to perform observations?*

•      *Does the learning climate play a role in performing observations?*

[Barriers and facilitators for the resident as estimated by the supervisor]

•       *Do you think the resident experiences any barriers to be observed?*

•        *How does your resident experience your presence when you observe him/her from your perspective?*

•        *Does the way your resident handles observations have an effect on you performing observations?*

•        *Are there factors that promote or make it easier for your resident to be observed during training?*

6. [Effects of performing observations on supervisor-resident relationship]

•         *Does performing observations affect the working relationship between you and your resident during the course?*

**Part III. Interview with supervisor-resident dyad**

1. [Use of observations by the dyad; current approach, development over time, communication and alignment]

At the start of the dyad interview, supervisor and resident are asked to summarise how they are using observations currently and *how* the*ir* *approach to using* observations developed over the course of the training year.

If not self-appointed, the following themes will be put forward:

o What is the object of observation

o Methods, initiative, planning and purpose of observation

o Role of learning question

o Mutual merits

o Barriers and facilitators

o Role of learning climate

Probing questions:

1A. Approach to and communication about performing observations and current use in clinical practice

•         *How do you currently use observations in training practice? Could you elaborate on this?*

•         *How do you communicate about your approach to use observations in training practice?*

If communicated explicitly:

•         *How is your approach actually in practice? Could you elaborate on this and provide examples?*

If not communicated explicitly:

•       *For what reason you don't communicate explicitly about performing observations?*

•       *When you do not communicate explicitly, do you come to a mutual approach or working arrangement on the use of observations in training practice? If yes, how?*

1B. Development of the use of observations over time

•         *If you compare the current approach of performing observations with the approach at the beginning of the training year, are there any differences?*

o *If so, how was the approach of observing in training practice at the beginning of this training year and how is it now?*

•         *How was the approach to performing observations 3 months after the start of the training year? Could you describe how you performed observations?*

o *How did it differ from the approach at the beginning of the training year?*

•         *How was the approach to observing six months after the start of the training year?*

o *How did it differ from the approach at the beginning of the training year?*

The interviewer provides a summary of the current use of observations and the development of the use of observations over the training year and asks if this summary is correct.

2. [Differences between supervisor and resident when establishing a working repertoire/ alliance regarding the use of observations]

•         *Have there been any differences in viewpoint or preference regarding the use of observations during training?*

If there were differences

•        *What were these differences?*

•        *How did you deal with these differences? And, more specifically, how did you communicate about these differences?*

If there were no differences

•         *For what reason do you think there were no differences? Did you communicate about the fact that there were no differences? How did you do that?*

 3. [Alignment and establishing a working repertoire]

•      *Do you feel that you have reached alignment on how to approach the use of observations?*

If alignment was reached

•         *What does this alignment look like? What did you agree on? Could you give examples of this?*

•         *How did you reach this alignment?*

If no alignment was reached

•          *For what reasons do you feel you did not reach alignment on the use of observations?*

•         *Did you have different preferences regarding the use of observations? On what points did you disagree?*

•         *How did you come to a working repertoire without alignment?*
