# Supplementary material for: Supervisory dyads’ communication and alignment regarding the use of workplace-based observations: a qualitative study in general practice residency
Source: BMC Med Educ. 2022 Apr 28;22:330. doi: 10.1186/s12909-022-03395-7 (PMC9052511; doi:10.1186/s12909-022-03395-7)
Supplement: Supplementary file 2 — Additional file 2. Overview of final template for data analysis. [file 12909_2022_3395_MOESM2_ESM.docx]

***Additional file 2.***

Overview of final template for data analysis

1. Goals of performing observations
   1. Monitor competence development
      1. Warrant patient safety
      2. Provide/gain feedback
   2. Prepare for assessment
2. Approach to performing observations
   1. Procedures
      1. Methods
      2. Setting
      3. Planning
      4. Frequency
   2. Triggers of and barriers for performing observations
      1. Time available
      2. Strive for resident autonomy
      3. Suboptimal performance
      4. External assessment
3. Dyads’ communication about the working repertoire and respective alignment
   1. Communication
      1. Explicitly discussed
      2. Not explicitly discussed
   2. Initiative and responsibility
